# A questionnaire development to assess the social representation of nurses in the Basque Country: a psychometric assessment

**DOI:** 10.7717/peerj.13903

**Published:** 2022-09-23

**Authors:** Verónica Tíscar-González, Leire Iturregui-Mardaras, Eztizen Miranda-Bernabé, Cristina Bermúdez-Ampudia, Maria Ángeles Cidoncha-Moreno, Sendoa Ballesteros-Peña

**Affiliations:** 1 Bizkaia Health Research Institute, Osakidetza, Bilbao Basurto Integrated Health Organization, University of the Basque Country, Academy of Nursing Sciences of Bizkaia, Bilbao, Spain; 2 University of the Basque Country, Leioa, Spain; 3 Bioaraba Health Research Institute, Vitoria, Spain; 4 Bioaraba Health Research Institute, General Head Office of Osakidetza, Basque Health Service, Subdirection of Nursing, Vitoria, Spain; 5 Biocruces Bizkaia Health Research Institute, Osakidetza, Santa Marina Hospital, University of the Basque Country, Academy of Nursing Sciences of Bizkaia, Bilbao, Spain

**Keywords:** Nursing, Journalism, Social, Nursing now

## Abstract

**Background:**

Only a few Spanish studies have explored how nurses are seen by society and no validated tools exist for this purpose in the scientific literature.

**Objectives:**

The aim was create and evaluate the psychometric characteristics of a questionnaire that explored the social representation of nursing in social and health care.

**Methods:**

Qualitative and quantitative methods were used to develop the questionnaire and the first version was created based on existing studies. A three-round Delphi technique was used that involved nurses, doctors, journalists and a politician. The pilot questionnaire was then tested and retested with 23 journalism students, with an interval of 10–14 days between the two phases. After further modifications, the questionnaire was sent to all the third and fourth year journalism students at the University of the Basque Country. Principal component factor analysis was used to identify the key components for the questionnaire.

**Results:**

A total of 141 third and fourth year journalism students took part in the study. The internal consistency of the 43-item perception section of the questionnaire had a Cronbach’s alpha value of 0.90. The 42.7% agreed or strongly agreed that nursing was an eminently scientific profession and 26.3% agreed or strongly agreed with the statement that nurses were presented in the media as health educators and disseminators. Just under a fifth (19.9%) agreed with the statement that society was aware of the competencies that nurses required.

**Conclusions:**

The questionnaire provided the first validated tool that allowed researchers to assess how nursing, and all of its areas of professional development, were perceived by society. This could enable studies to assess the evolution of the profession over time and between different socio-cultural contexts.

## Introduction

In June 2021, the international Nightingale Challenge was renamed Nursing Now. Led by Coventry University, UK and The Burdett Trust for Nursing, it is working with health employers around the world to create leadership development opportunities for 100,000 nurses and midwives in more than 150 countries by the end of 2022. The Nursing Now movement has undoubtedly been a turning point in the history of nursing, as it promotes the profession. It has also collaborated in the development of health policies (World Health Organization, 2018).

One of the objectives of the campaign has been to promote and empower the political role of nurses, based on the results of the Triple Impact of Nursing Report, which was published by the UK (All-Party Parliamentary Group on Global Health, 2016). The report’s authors concluded that the empowerment and political participation of nurses has an impact on gender equality and the economy of countries in addition to improving the health of the population and and contribute to the social visibility of nursing.

The image and social identity of a profession is determined by how it is perceived by society (Fernández Gutiérrez, 2017) and the media play a key role in this process. This research recognised the media’s ability to shape public opinion (Lippmann, 1922), as they determine the issues that citizens talk about and reflect on. Their role in shaping the citizens’ agenda was outlined by McCombs & Shaw (1972) in their agenda setting theory. A later study stated that journalists can determine what news events are reported and how they are presented, in accordance with framing theory (McCombs & Ghanem, 2001).

However, the coverage that journalists provide on health issues has rarely been investigated by academic researchers (Wallington et al., 2010). Several studies have revealed that nurses are rarely featured in health reports and can even seem invisible (Mason et al., 2018).

Despite the advances made by the nursing profession in recent decades, including academic developments, its social image continues to be plagued by stereotypes (Harmes, Harmes & Harmes, 2021). A study by Bogdan & Gurylina (2019) analysed nursing images in social media and demonstrated the existence of four clear stereotypes: guardian angel, doctor’s assistant, authoritarian nurse and sensual nurse. All of them have negative connotations and are far removed from the real life roles and competencies of nurses. A critical review concluded that the media needed to be harnessed to present society with an image of nurses that was more in line with reality (Gill & Baker, 2019). Nursing is a largely female-dominated profession in a male-dominated media industry, which may explain why the social image has not kept pace with the advancement of the profession (Gill & Baker, 2021).

Very few Spanish studies have directly addressed how nurses are seen by society. One exception was Heierle Valero (2009), who analysed the images of nurses published in the newspaper *El País* between 2004 and 2006. The results showed that nursing had little impact on this medium and that it was seen as a subordinate profession without its own identity. The study identified the need to improve how the identity of nurses was projected in society, in order to promote nursing and gain recognition for the profession.

Another later study, carried out in 2013, explored what journalism degree students knew about nursing. The results were disappointing, as students were generally unaware of the wide range of competencies that nurses can offer (Recuero Vázquez et al., 2014). Although there are other validated questionnaires in the scientific literature that explore the social vision of the nurse (Toth, Dobratz & Boni, 1998; Cukljek, Juresa & Babic, 2017), we did not find in the literature any questionnaire validated in Spanish, and we considered necessary to create a new tool that would thoroughly evaluate all possible areas of development: management and health policy, teaching, clinical practice and research.

We, therefore, considered it necessary to delve deeper into how society saw nurses and enable us to evaluate the evolution of the profession and its development. This included the real impact of the COVID-19 pandemic on how society perceived nurses. We wanted to see whether this global pandemic highlighted the important role that the nursing profession has played in tackling a major public health problem. This included any recognition of the need for nurses to be empowered so that they could participate in health policies (Catton, 2020).

The media play a key role in making nurses visible to society and in highlighting the real scope of the nursing profession (Kress et al., 2018). That is why we wanted to explore the perceptions of journalism students. As the journalists of tomorrow, they will have the power to portray the real image of nursing or to perpetuate stereotypical images that are far removed from the real competencies of today’s nurses.

### Aims

The aims of this study were to create and evaluate the psychometric characteristics of a questionnaire that assessed how society sees nurses involved in delivery of health and social care. To do this, we explored how nurses were perceived by third and fourth year journalism degree students at the public University of the Basque Country, Spain.

## Methodology

### Design

**Figure 1:**
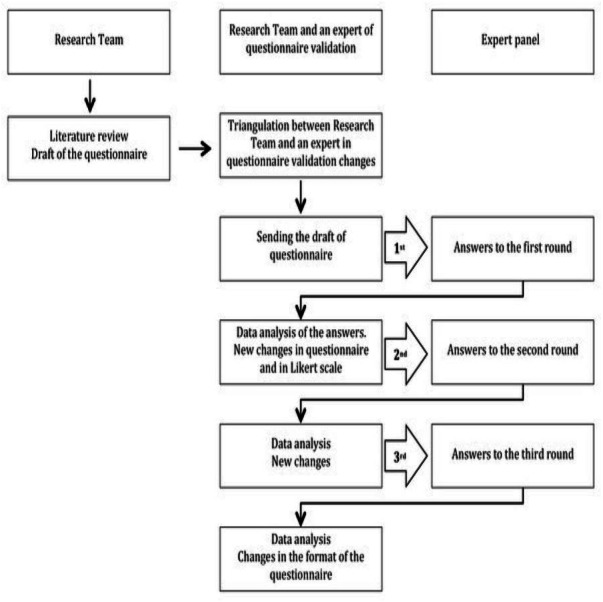
The Delphi technique.

The questionnaire was developed and evaluated using quantitative and qualitative methods (Polit & Beck, 2012). The first version of the survey was prepared by two researchers from the nursing area. A literature search was carried out to identify the topics to be evaluated and the specific items were defined according to the literature. Content validation was performed sequentially. All the researchers reviewed the initial questionnaire and then a group of 10 experts carried out a critical analysis using the Delphi method (Landeta, 2006; Yañez & Cuadra, 2008). This expert panel comprised Spanish nurses and physicians with experience of research and/or management, health policy and journalists. A total of three rounds were conducted (Fig. 1). The zero version of the questionnaire had 43 items, and after the three rounds of the Delphi technique the questionnaire underwent changes in terms of content and number of items. There were a total of 48 items in the final version.

After the last critical analysis round, we held a pilot trial to check that the questionnaire was clear and easy to understand. The participants were 23 third and fourth year journalism degree students who were selected by convenience sampling. We then collected the feedback from these participants, as well as the expert group and those in charge of the data collection. The definitive questionnaire was finalised after a number of changes for clarity.

### Participants

All 424 students enrolled in the third and fourth years of Degree in Journalism at the University of the Basque Country, Spain, during the 2020/21 academic year were invited to voluntarily participate in the study. The questionnaire was distributed by email between November and December 2020, at the end of the classes at the University.

### Measures and statistical methods

The final questionnaire comprised a section on socio-demographic variables, a series of 48 closed-ended questions that required *yes, no* or *don’t know* answers and a series of questions that used a six-point Likert scale to assess the degree of agreement or conformity to different statements. The evaluations ranged from zero (strongly disagree) to five (strongly agree) and explored eight dimensions. The scores for each item were added together to obtain a score for each factor.

The reliability and stability of the final questionnaire over time was assessed by applying a test-retest protocol to the subjects that participated in the pilot trial, with an interval of 10–14 days between the two phases. The level of concordance (agreement) for each item was determined by using Cohen’s Kappa coefficient (*k* = 0.514; *p* < 0.001) and the reliability of the items that were evaluated by the Likert scale were calculated using the intraclass correlation coefficient, which was 0.774 (*p* < 0.001). The anonymity of the questionnaire was maintained by using paired codes based on the last two digits and letter of each subject’s national identity card.

### Statistical analysis

Absolute numbers and percentages were used to describe the characteristics of the qualitative variables and means and standard deviations (SD) were used for the quantitative variables.

The Student’s *t*-test for independent samples was used to compare the quantitative variables between the two groups, as these followed a normal distribution.

Reliability analysis was used to assess the internal consistency of the questionnaire and this was carried out by determining the Cronbach’s alpha coefficients, with values of more than 0.7 representing good reliability.

### Factor analysis

The questionnaire items were reduced by carrying out an exploratory factor analysis on the principal components. The applicability assumptions of the factor analysis were tested using the Kaise-Meyer-Olkin index (KMO) and Bartlett’s test of sphericity. An appropriate value for the KMO Index, which compared the observed correlation coefficients with the partial correlation coefficients was considered to be between 0.5 and 1.0. The KMO that we obtained was 0.807. Bartlett’s test of sphericity allowed us to check that the correlation matrix was an identity matrix. The significance required was <0.05 and ours was <0.001. Therefore, the assumptions for the factor analysis were met.

The factorial rotation procedure was then carried out on the components, under Varimax rotation, to maximise the independence between the factors and facilitate their interpretation.

The anti-image matrix was examined to obtain the final factorial model. Any values close to one were included and any with a low score, were eliminated. We also eliminated any items that did not meet the first assumption of a factor, because there were at least two correlated items.

The statistical treatment of the data was carried out using SPSS, version 23 (IBM Corp, NY, USA) and R 3.5.0 (R Foundation, Vienna, Austria). Statistical significance was considered when the bilateral *p*-value was <0.05.

### Ethical considerations

It was considered that by answering the questionnaire voluntarily and completely anonymously they agreed to participate in the study. This study was approved by the Ethics Committee of the University of the Basque Country (code M10_2019_312). All participants gave their informed consent and confidentiality was guaranteed and achieved.

## Results

The Nursing Social Representation (NSR) questionnaire was created and modified according to the three Delphi rounds and the results of the pilot study. It is composed of a total of 48 items.

The response rate was of 33.2%, a third of the possible study population enrolled in the study and the cohort comprised 141 third and fourth year students (57.4% female), with a mean age of 21.8 ± 3.15 years. Of these 141 students, 89.4% had completed their high school studies. (In Spain, access to university is also possible in some cases through higher vocational training, not only through baccalaureate studies). The majority (87.9%) had been seen by at least one nurse in the last two years and 94.3% stated that someone in their immediate student circle had been seen by at least one nurse in the last two years (Table 1).

**Table 1:** Demographics/social characteristics.

**Demographics and social related characteristics of the 141 participants**	
**Demographics**	
**Sex** Female number (%)	81 (57.4%)
**Age** (mean, standard deviation)	21.8 ±3.15
**Social and work related characteristics**	
**Previous studies**	
Bachelor’s degree	126 (89.4%)
Professional degree	6 (4.3%)
Other undergraduate studies or equivalent	9 (6.4%)
**Academic course**	
3rd year	34 (24.1%)
4th year	107 (75.9%)
**Has been attended by nurse(s) in the past two years**	
No	17 (12.1%)
Yes	124 (87.9%)
**Someone in their social circle is a nurse**	
No	46 (32.6%)
Yes	95 (67.4%)
**Someone in their environment been attended by nurses in the last two years**	
No	8 (5.7%)
Yes	133 (94.3%)

In relation to the students’ knowledge of nursing studies, 97.9% knew that nursing required a university degree, 80.1% knew that nurses could undergo postgraduate training, 62.4% that they could study for a doctorate; and 52.5% (73) that they could access specialities through a national competitive call for applications. In addition 70.9% believed that medical and surgical nursing was a speciality and 80.1% that considered emergency and critical care nursing was a specialty. However, none of these areas are currently nursing specialties in Spain (Table 2).

**Table 2:** Knowledge about nursing education.

Knowledge about nursing education	
01. Nursing is a degree in Spain	
No	2 (1.4%)
Don’t know	1 (0.7%)
Yes	138 (97.9%)
02. Nurses can access postgraduate studies	
No	3 (2.1%)
Don’t know	25 (17.7%)
Yes	113 (80.2%)
03. Nurses can access doctoral studies	
No	3 (2.1%)
Don’t know	50 (35.5%)
Yes	88 (62.4%)
04. Nurses can access specialties through a state-wide selection process	
No	5 (3.6%)
Don’t know	61 (43.9%)
Yes	73 (52.5%)
05. Do you consider the following to be nursing specialties?	
05a. Mental health nursing	
No	18 (12.8%)
Don’t know	28 (19.8%)
Yes	95 (67.4%)
05b. Paediatric nursing	
No	6 (4.3%)
Don’t know	14 (10.1%)
Yes	119 (85.6%)
05c. Family and community nursing	
No	17 (12.1%)
Don’t know	39 (27.7%)
Yes	84 (59.6%)
05d. Occupational health nursing	
No	27 (12.1%)
Don’t know	48 (34%)
Yes	66 (46.8%)
05e. Geriatric nursing	
No	3 (2.2%)
Don’t know	16 (11.4%)
Yes	121 (86.4%)
05f. Midwifery	
No	13 (9.3%)
Don’t know	15 (10.7%)
Yes	112 (80.0%)
05g. Medical and surgical care nursing	
No	14 (9.9%)
Don’t know	27 (19.2%)
Yes	100 (70.9%)
05h. Emergency and urgency nursing	
No	14 (9.9%)
Don’t know	14 (9.9%)
Yes	113 (80.2%)
05i. Intensive care nursing	
No	10 (7.1%)
Don’t know	18 (12.8%)
Yes	113 (80.1%)

We found that 42.7% agreed or strongly agreed that nursing was an eminently scientific profession and 26.3% agreed or strongly agreed with the statement that nurses were presented in the media as health educators and disseminators. Just under a fifth (19.9%) agreed with the statement that society was aware of the competencies that nurses required (Table 3).

**Table 3:** Slightly agree and strongly agree *n*%.

	**0**	**1**	**2**	**3**	**4**	**5**
Item 6. Nursing is a profession that can be practised by both men and women	1 (0.71%)	–	–	–	3 (2.13%)	137 (97.2%)
Item 7. The social visibility of nurses is independent of the gender of the profession.	14 (9.9%)	26 (18.4%)	21 (14.9%)	27 (19.1%)	21 (14.9%)	32 (22.7%)
Item 8. Proportionally, there are more male nurses than female nurses in senior positions of responsibility.	17 (12.2%)	12 (8.63%)	34 (24.5%)	31 (22.3%)	32 (23.0%)	13 (9.35%)
Item 9. Proportionally, there are more men than women in teaching and research positions in care.	9 (6.43%)	13 (9.29%)	29 (20.7%)	44 (31.4%)	34 (24.3%)	11 (7.86%)
Item 10. Nurses have autonomy in decision-making about the care of their patients.	9 (6.38%)	18 (12.8%)	41 (29.1%)	41 (29.1%)	24 (17.0%)	24 (17.0%)
Item 11. Nurses are competent to detect or diagnose the health problems and care needs of individuals.	4 (2.90%)	18 (13.0%)	9 (6.52%)	26 (18.8%)	43 (31.2%)	38 (27.5%)
Item 12. Nurses can autonomously treat minor illnesses of a self-limiting nature.	5 (3.60%)	5 (3.60%)	13 (9.35%)	29 (20.9%)	47 (33.8%)	40 (28.8%)
Item 13. Nurses are able to independently prescribe over-the-counter medicines.	25 (17.9%)	20 (14.3%)	22 (15.7%)	29 (20.7%)	24 (17.1%)	20 (14.3%)
Item 14. Nurses also work in coordination with other members of the health team to respond to the needs of patients.	–	1 (0.71%)	4 (2.86%)	17 (12.1%)	36 (25.7%)	82 (58.6%)
Item 15. Nursing assistants (*i.e.*, nurses who provide direct care to people) perform managerial and administrative tasks in their daily clinical practice.	1 (0.71%)	8 (5.71%)	21 (15.0%)	36 (25.7%)	42 (30.0%)	32 (22.9%)
Item 16. Any trained health professional should be able to have access to a senior management position within an institution (e.g., management of a health organisation, health councils...) regardless of their profession.	7 (5.11%)	6 (4.38%)	12 (8.76%)	28 (20.4%)	42 (30.7%)	42 (30.7%)
Item 17. Any trained health professional should be able to participate in the development of health policies at any state level, regardless of whether they are male or female.	5 (3.68%)	3 (2.21%)	3 (2.21%)	10 (7.35%)	22 (16.2%)	93 (68.4%)
Item 18. The nursing profession is eminently scientific.	4 (2.94%)	13 (9.56%)	16 (11.8%)	45 (33.1%)	31 (22.8%)	27 (19.9%)
Item 19. Research is part of the competencies of nurses.	2 (1.48%)	9 (6.67%)	14 (10.4%)	39 (28.9%)	35 (25.9%)	36 (26.7%)
Item 20. Journals focused on care are recognised for their prestige among scientific publications (impact factor).	3 (2.21%)	12 (8.82%)	32 (23.5%)	42 (30.9%)	41 (30.1%)	6 (4.41%)
Item 21. The results of research carried out by nurses aim to improve the health of individuals and their communities.	–	1 (0.73%)	6 (4.38%)	17 (12.4%)	54 (39.4%)	59 (43.1%)
Item 22. The results of research conducted by nurses can promote the development of health policies.	1 (0.74%)	2 (1.47%)	5 (3.68%)	22 (16.2%)	48 (35.3%)	58 (42.6%)
Item 23. The results of research conducted by nurses can contribute to the sustainability of the healthcare system.	–	1 (0.74%)	2 (1.47%)	28 (20.6%)	46 (33.8%)	59 (43.4%)
Item 24. The research carried out by nurses include the field of healthcare.	–	–	9 (6.67%)	31 (23.0%)	52 (38.5%)	43 (31.9%)
Item 25. The research carried out by nurses include public health problems.	–	4 (2.92%)	8 (5.84%)	32 (23.4%)	42 (30.7%)	51 (37.2%)
Item 26. The research carried out by nurses include gender inequalities and social determinants of health.	2 (1.48%)	4 (2.96%)	10 (7.41%)	34 (25.2%)	41 (30.4%)	44 (32.6%)
Item 27. Nurses can carry out research into many possible fields of research.	–	–	11 (8.09%)	24 (17.6%)	44 (32.4%)	57 (41.9%)
Item 28. Nurse educators develop their competence at university level.	6 (4.38%)	10 (7.30%)	10 (7.30%)	36 (26.3%)	46 (33.6%)	25 (18.2%)
Item 29. Nurses can become university lecturers.	2 (1.46%)	3 (2.19%)	3 (2.19%)	18 (13.1%)	32 (23.4%)	79 (57.7%)
Item 30. Nurses develop teaching competencies in clinical settings.	2 (1.46%)	2 (1.46%)	2 (1.46%)	36 (26.3%)	53 (38.7%)	32 (23.4%)
Item 31. Nurses engage with the media as health educators and communicators..	15 (10.9%)	28 (20.4%)	25 (18.2%)	33 (24.1%)	24 (17.5%)	12 (8.76%)
Item 32. Nurses are visible in social networks as disseminators and educators in health matters.	14 (10.2%)	22 (16.1%)	27 (19.7%)	32 (23.4%)	25 (18.2%)	17 (12.4%)
Item 33. The media has a close relationship with the professional nursing associations.	8 (5.84%)	27 (19.7%)	41 (29.9%)	26 (19.0%)	27 (19.7%)	8 (5.84%)
Item 34. Nurses are accessible and trained to respond immediately to the media when there are emergencies and health alerts.	7 (5.11%)	13 (9.49%)	18 (13.1%)	35 (25.5%)	39 (28.5%)	25 (18.2%)
Item 35. Nurses provide the media with accurate and verified sources of information.	2 (1.46%)	2 (1.46%)	13 (9.49%)	37 (27.0%)	52 (38.0%)	31 (22.6%)
Item 36. The competences of nurses are known at the societal level.	13 (9.56%)	25 (18.4%)	40 (29.4%)	31 (22.8%)	19 (14.0%)	8 (5.88%)
Item 37. The competencies of nurses include promoting the health of individuals and his or her community.	2 (1.47%)	2 (1.47%)	15 (11.0%)	43 (31.6%)	47 (34.6%)	27 (19.9%)
Item 38. Nurses play a key role in preventing disease in the community.	1 (0.73%)	1 (0.73%)	8 (5.84%)	30 (21.9%)	46 (33.6%)	51 (37.2%)
Item 39. Nurses play a key role in the community by providing telephone health advice services.	1 (0.74%)	5 (3.70%)	10 (7.41%)	25 (18.5%)	43 (31.9%)	51 (37.8%)
Item 40. Telemonitoring of complex patients is an area of development for nurses.	2 (1.47%)	4 (2.94%)	19 (14.0%)	44 (32.4%)	46 (33.8%)	21 (15.4%)
Item 41. Nurses are trained and knowledgeable professionals who can ensure people have proper health education.	–	2 (1.46%)	3 (2.19%)	21 (15.3%)	46 (33.6%)	65 (47.4%)
Item 42. Nurses are good health outreach workers.	1 (0.74%)	2 (1.47%)	5 (3.68%)	39 (28.7%)	45 (33.1%)	44 (32.4%)
Item 43. You are familiar with aspects of the nursing profession through traditional media.	15 (10.9%)	25 (18.2%)	31 (22.6%)	31 (22.6%)	25 (18.2%)	10 (7.30%)
Item 44. The results of nursing research are also shared with society through the media.	14 (10.2%)	25 (18.2%)	28 (20.4%)	48 (35.0%)	15 (10.9%)	7 (5.11%)
Item 45. Your perception of nurses has improved after the COVID-19 pandemic.	4 (2.94%)	2 (1.47%)	4 (2.94%)	16 (11.8%)	50 (36.8%)	60 (44.1%)
Item 46. Nurses have shown decisive management in helping to resolve the COVID-19 pandemic.	1 (0.73%)	1 (0.73%)	5 (3.65%)	11 (8.03%)	24 (17.5%)	95 (69.3%)
Item 47. The COVID-19 pandemic crisis has contributed to raising the visibility of the research competencies of nurses.	2 (1.46%)	7 (5.11%)	10 (7.30%)	17 (12.4%)	41 (29.9%)	60 (43.8%)
Item 48. There should be a nurse figure in the COVID-19 pandemic reconstruction commission.	–	2 (1.46%)	1 (0.73%)	11 (8.03%)	27 (19.7%)	96 (70.1%)

Table 4 shows the rotated component matrix and the eight dimensions that made up the questionnaire (Prevention, health promotion and health policies; Social visibility; Improvement in the social visibility of the profession during the COVID-19 pandemic; Research in care, social determinants and public health; Autonomy in decision-making in clinical practice; Nursing in the university setting; Scientific dissemination and gender perspective). The mean value of the prevention, health promotion and health policies dimension was 3.91 and it was 2.41 for the social visibility dimension. No statistically significant differences were found when the socio-demographic differences of the students were taken into account. The mean value of the social visibility of the profession during the COVID-19 pandemic was 4.27.

**Table 4:** Rotated component matrix.

	1	2	3	4	5	6	7	8		
Q38_Preventing illness	0.798									
Q37_Health promotion	0.780									
Q39_Health policies	0.775									
Q42_Scientific dissemination agents	0.730									
Q41_Health education	0.682									
Q23_Sustainability of the healthcare system	0.663									
Q21_Impact on health improvement	0.651									
Q35_Media and truthful information	0.567									
Q34_Media and sanitary alarm	0.564									
Q27_ Multiple fields of research	0.416									
Q44_ Research results nurse society		0.872								
Q43_ Media and communication on nursing activity		0.774								
Q36_ Socially known competencies		0.644								
Q31_ Media disseminators		0.570								
Q32_ Visibility in social networks		0.515								
Q45_ Improved perception of nurses			0.865							
Q46_ Decisive management work			0.744							
Q48_ Pandemic reconstruction			0.646							
Q47_ Making research skills on crises visible			0.504							
Q24_ Research in care				0.801						
Q25_ Public health problems				0.771						
Q26_ Gender inequalities				0.524						
Q13_ Prescribing autonomously					0.801					
Q10_ Autonomy in decision making					0.488					
Q11_ Competent decision-makers					0.487					
Q12_ Autonomous self-limiting diseases					0.437					
Q28_ University environment						0.689				
Q17_Health policies development						0.664				
Q29_ University professors						0.605				
Q21_ Scientific publications							0.742			
Q19_ Research							0.497			
Q9_ More men teaching								0.829		
Q8_ More men in positions of responsibility								0.804		
Q14_ Working in a coordinated way										
Q40_Telemonitoring										
Q30_ Clinical teacher										

When it came to the research in care, social determinants and the public health domain, the mean value was 3.89 (SD: 0.86) and statistically significant differences were found (*t* = −2.743; *p* = 0.007) between the mean and range values for men 3.65 (SD: 0.91) and women 4.06 (SD: 0.79). This indicated that women had better perceptions than men.

Statistically significant differences were found in the research in care, social determinants and public health domain (*t* = −2.135; *p* = 0.035). Students who had friends or family members who were nurses had better mean and range scores for perceptions 4.0 (SD: 0.86) than those who did not 3.67 (SD: 0.84). This personal connection with nursing also led to statistically significant differences in the scientific dissemination domain (*t* = −3.261; *p* = 0.001). The overall mean value was 3.21 (0.96) but students who knew nurses had a better perception of this domain 3.40 (0.90) than those who did not 2.84 (0.98).

The mean value of the autonomy in decision-making in clinical practice domain was 3.0 (SD: 1.03) and for university nursing it was 3.99 (SD: 0.93).

Finally, the mean value for gender perspective was 2.73 (SD:1.21). The database is available at Mendeley (Tíscar-González et al., 2021).

### Psychometric characteristics

The internal consistency of the 43-item perception section of the questionnaire had a Cronbach’s alpha of 0.90. The reduction in the scales was assessed using principal component factor analysis and this led to eight factors. The common factors and the component matrix of the principal component analysis are summarized in Table 4. Table 5 shows the Cronbach’s Alphas for each factor.

**Table 5:** Factors’ Cronbach’s alpha value.

Factor	**Items**	**Cronbach’s Alpha value**
Factor 1: Prevention, health promotion and health policies	Q16, Q17, Q18, Q22, Q29, Q30, Q32, Q33, Q34, Q36, Q37	0.908
Factor 2: Social visibility	Q26, Q27, Q31, Q38, Q39	0.815
Factor 3: Improvement in the social visibility of the profession during the COVID-19 pandemic	Q40, Q41, Q42, Q43	0.767
Factor 4: Research in care, social determinants and public health	Q19, Q20, Q21	0.763
Factor 5: Autonomy in decision-making in clinical practice	Q5, Q6, Q7, Q8	0.703
Factor 6: Nursing in the university setting	Q12, Q23, Q24	0.642
Factor 7: Scientific dissemination	Q14, Q15	0.451
Factor 8: Gender perspective	Q3, Q4	0.690

## Discussion

This study developed and validated a 43-item questionnaire, with high reliability, to assess how undergraduate journalism students at the public University of the Basque Country, Spain perceived the social representation of nursing. This was an important target audience, as these students are the journalists of the future and their articles and news reports will help to drive public opinion. Training journalists is one of the elements that influences the news making process (Shoemaker & Reese, 1996), as these future communicators will portray, and influence, the image that society has of nurses. The participants were young adults, but some of them had experienced contact with nurses in the last two years, which had given them an updated view of the profession.

A 2013 study of journalism degree students (Recuero Vázquez et al., 2014), which used a different tool, showed that they were unaware of the functions that nurses could perform and the level of education that was required for a nursing career. This was also seen in our study.

The amount of knowledge that the journalism students had about nursing education decreased as the level of the nursing qualifications increased. For example, more than a third of those surveyed did not know that nurses could study for a doctorate and more than half did not know that there was a selective national process for nurse specialists. Some also lacked knowledge about the various areas that nurses could specialize in. This lack of knowledge was in line with the image that the media presents of nurses (Heierle Valero, 2009). There has been misinformation and confusion and the profession has a poor image. For example nurses have been perceived to have little professional independence and be subordinate to doctors, especially if they are female. This means that nursing has not been seen as a very attractive and visible career (Errasti-Ibarrondo, Arantzamendi-Solabarrieta & Canga-Armayor, 2012; Sánchez-Gras, 2017).

Improvements in the social visibility of the profession during the COVID-19 pandemic was the most highly valued factor, as the media saw nurses as sources of information and as subjects of information (DeWees & Miller, 2020). Their presence in social networks has also increased during the pandemic (Forte & Pires, 2020) and so has information about their contribution to healthcare. That could be why this factor was one that was most valued in this study. The COVID-19 pandemic may be an opportunity to promote the global visibility of nursing (Torres Contreras, 2020), by showing their skills and care competencies, their knowledge and how they carry out holistic and integrative research. On the other hand, this study coincides in time with the nursing now campaign, which aims to make the potential of nurses socially visible (World Health Organization, 2018).

However, the lowest rated factors in this study were social visibility, the gender perspective, as in female nurses being subordinate to physicians, and autonomy in decision making. These confirmed the finding of other studies that nursing has had a poor professional identity and that nurses have been perceived to have limited professional autonomy (Errasti-Ibarrondo, Arantzamendi-Solabarrieta & Canga-Armayor, 2012; Franco Coffré, 2020; Gill & Baker, 2019; Sánchez-Gras, 2017).

As early as 2007, Kemmer & Paes da Silva pointed out that journalists lacked knowledge about nursing, the labour market and professional categorisation. That study highlighted the need to establish strategies to construct a more coherent image that valued the potential of the nursing profession.

The media play a fundamental role in how society sees nurses (Happer & Philo, 2013). Gill & Baker (2019) also emphasised the importance of working with the media to create a more accurate image of how society sees the roles and competencies of the nursing profession and make their real contribution to healthcare more visible. Ensuring that journalists are more aware of the contribution that nurses make to society would be a good first step, as it modifies the image projected by the media.

The main limitation of this exploratory study was that it was limited to journalism students from the University of the Basque Country. It would be interesting if future studies could focus on students from other regions of the country, as well as the general population.

The strength of this study is that an optimal cronbach’s alpha value is achieved with a value of 0.90 (Campbell, 1976).

Having a validated tool that can assesses the how society sees the nursing profession could be very useful for evaluating its future development and comparing different socio-cultural contexts. On the other hand, it would be interesting to incorporate within the journalism degree, meeting points and seminars where nurses can share their experience and the results of research in care, and explain the different roles. It would also be useful to carry out practical exercises in the classroom, such as the presentation of published information on the nursing profession in which students must detect errors (such as the dissemination of stereotypes or the subordination of the nursing profession); at the end of the exercise, propose a debate in the classroom. This would contribute to better training of future journalists, in line with Sustainable Development Goals 3 (health and well-being), 4 (quality education) and 5 (gender equality). In addition, training and sensitisation of journalism students would likely reduce the spread of stereotypes in reporting on the nursing profession, leading to greater social awareness of the contribution of nurses in the field of health and progress towards achieving a better informed public opinion.

## Conclusions

This study, which was aimed at journalism students, provides a validated tool with which to assess nurse social representation in all areas of professional development with an optimal internal consistency. It will enable researchers to measure the evolution of the profession over time and in different societies.

The perception of the nursing profession that was presented by the journalism students who took part in this study was mainly limited to the field of nursing care. In general, they were unaware of the possible academic development of nursing, as well as the contribution that nurses play in areas such as research or management. This study highlights the need to improve how society sees the impact that the nursing profession has on healthcare systems and on the health of the population.

##  Supplemental Information

10.7717/peerj.13903/supp-1 Supplemental Information 1 Questionnaire in Spanish: original version

10.7717/peerj.13903/supp-2 Supplemental Information 2 Raw data

10.7717/peerj.13903/supp-3 Supplemental Information 3 English questionnaire

## Additional Information and Declarations

### Data Availability

The following information was supplied regarding data availability: Raw data are available in the Supplementary Files.

## References

[ref-1] All-Party Parliamentary Group on Global Health (2016). Triple Impacto. Cómo el desarrollo de la enfermería mejorará la salud, promoverá la igualdad de género y apoyará el crecimiento económico. Global Health.

[ref-2] Bogdan I, Gurylina M (2019). Four big stereotypes on nurses in mass consciousness: on the materials of the Moscow people opinion analysis in social media. Problems of Social Hygiene, Public Health and History of Medicine.

[ref-3] Campbell JP (1976). Psychometric theory. Handbook of industrial and organizational psychology.

[ref-4] Catton H (2020). Nursing in the COVID-19 pandemic and beyond: protecting, saving, supporting and honouring nurse. Nursing and Health Policy Perspectives.

[ref-5] Cukljek S, Juresa V, Babic J (2017). The cross-cultural (transcultural) adaptation and validation of the nursing image questionnaire. Nurse Education Today.

[ref-6] DeWees MA, Miller AC (2020). The costs of care: a content analysis of female nurses’ media visibility and voices in the United States, China, and India during the COVID-19 pandemic. COVID-19.

[ref-7] Errasti-Ibarrondo B, Arantzamendi-Solabarrieta M, Canga-Armayor N (2012). La imagen social de la enfermería: una profesión a conocer. Anales Del Sistema Sanitario de Navarra.

[ref-8] Fernández Gutiérrez DÁ (2017). Por qué su imagen profesional puede afectar seriamente a la salud de las personas que atiende (y a la suya propia). ENE, Revista de Enfermeria.

[ref-9] Forte E, Pires D (2020). Nursing appeals on social media in times of coronavirus. Revista Brasileira de Enfermagem.

[ref-10] Franco Coffré J (2020). Percepción social de la profesión de enfermería. Revista Enfermería Actaul En Costa Rica.

[ref-11] Gill J, Baker C (2019). The power of mass media and feminism in the evolution of nursing’s image: a critical review of the literature and implications for nursing practice. Journal of Medical Humanities.

[ref-12] Gill J, Baker C (2021). The power of mass media and feminism in the evolution of nursing’s image: a critical review of the literature and implications for nursing practice. Journal of Medical Humanities.

[ref-13] Happer C, Philo G (2013). The role of the media in the construction of public belief and social change. Journal of Social and Political Psychology.

[ref-14] Harmes MK, Harmes B, Harmes MA (2021). The nurse in popular media: critical essays.

[ref-15] Heierle Valero C (2009). La imagen de la enfermera a través de los medios de comunicación de masas: La prensa escrita. Index de Enfermeria.

[ref-16] Kress D, Godack CA, Berwanger TL, Davidson PM (2018). The new script of nursing: using social media and advances in communication–to create a contemporary image of nursing. Contemporary Nurse.

[ref-17] Landeta J (2006). Current validity of the Delphi method in social sciences. Technological Forecasting and Social Change.

[ref-18] Lippmann W, Lippmann W (1922). The world outside and the pictures in our heads. Public opinion.

[ref-19] Mason DJ, Nixon L, Glickstein B, Han S, Westphaln K, Carter L (2018). The woodhull study revisited: nurses’ representation in health news media 20 years later. Journal of Nursing Scholarship.

[ref-20] McCombs M, Ghanem SI (2001). The convergence of agenda setting and framing. Framing public life.

[ref-21] McCombs ME, Shaw DL (1972). The Public Opinion Quarterly.

[ref-22] Polit DF, Beck CT (2012). Nursing research: generating and assessing evidence for nursing practice.

[ref-23] Recuero Vázquez M, Hontanaya RGómez, Hernández Gómez CI, Muñoz Rastrilla S (2014). Imagen social de las enfermeras: percepción de los futuros periodistas. Metas de Enferm.

[ref-24] Sánchez-Gras S (2017). Imagen de la enfermería a través de la prensa escrita ¿necesitamos visibilizar los cuidados enfermeros?. Cultura de Los Cuidados.

[ref-25] Shoemaker PJ, Reese SD (1996). Mediating the message.

[ref-26] Tíscar-González V, Iturregui-Mardaras L, Miranda E, Bermúdez-Ampudia C, Cidoncha-Moreno MA, Ballesteros-Peña S (2021). Database: a questionnaire development to assess the social representation of nurses in the Basque Country: a psychometric assessment. Mendeley Data.

[ref-27] Torres Contreras C (2020). COVID-19 Pandemics: an opportunity to give Nursing global visibility. Revista Gaúcha de Enfermagem.

[ref-28] Toth JC, Dobratz MA, Boni MS (1998). Attitude toward nursing of students earning a second degree and traditional baccalaureate students: are they different?. Nursing Outlook.

[ref-29] Wallington SF, Blake KD, Taylor-Clark K, Viswanath K (2010). Challenges in covering health disparities in local news media: an exploratory analysis assessing views of journalists. Journal of Community Health.

[ref-30] World Health Organization (2018). Nursing Now Campaign. https://www.who.int/news/item/27-02-2018-nursing-now-campaign.

[ref-31] Yañez R, Cuadra R (2008). La Tecnica Delphi Y La Investigacion En Los Servicios De Salud. Ciencia y Enfermeria.

