# Supplementary material for: A questionnaire development to assess the social representation of nurses in the Basque Country: a psychometric assessment
Source: PeerJ. 2022 Sep 23;10:e13903. doi: 10.7717/peerj.13903 (PMC9512000; doi:10.7717/peerj.13903)
Supplement: Supplemental Information 1 [file peerj-10-13903-s001.pdf]

Periodismo y Enfermería

Preguntas Respuestas 141 Configuración

Sección 1 de 3

REPRESENTACIÓN SOCIAL DE LAS ENFERMERAS POR ESTUDIANTES DE PERIODISMO

Muchas gracias por colaborar en esta encuesta. A continuación, te realizaremos diversas preguntas orientadas a explorar la representación social de la enfermería desde la perspectiva del alumnado del Grado de Periodismo.

Esta encuesta está enmarcada en el proyecto de investigación ENFERMERAS DESDE LA PERSPECTIVA DE ESTUDIANTES DE GRADO DE PERIODISMO DEL PAÍS VASCO, que cuenta con el informe favorable del Comité de Ética de la UPV/EHU, siendo sus investigadores principales Verónica Tíscar (veronica.tiscargonzales@osakidetza.eus), Leire Iturregui (leire.iturregui@ehu.eus), Eztizen Miranda (eztizen.miranda@ehu.eus) y Sendoa Ballesteros (sendoa.ballesteros@ehu.eus).

La encuesta es totalmente ANÓNIMA y VOLUNTARIA, por lo que te rogamos que seas lo más sincero/a posible con las respuestas. Completarla apenas te llevará 10 minutos.

Al cumplimentarla de forma voluntaria entenderemos tu consentimiento tácito para participar en la investigación. Recuerda que, una vez iniciada la encuesta, puedes retirarte y abandonar en cualquier momento, si así lo deseas.

Después de la sección 1 Ir a la siguiente sección

TUS DATOS SOCIO-DEMOGRAFICOS

Recuerda que estos datos son anónimos y su tratamiento es confidencial

Género

1. Mujer
2. Hombre
3. No binario

Fecha de nacimiento

Mes, día, año

Estudios previos

1. Bachillerato / LOGSE
2. FP / Ciclo Formativo
3. Otros estudios de grado o equivalente
4. Otros

Curso académico actual

1. 1º
2. 2º
3. 3º
4. [illegible]

¿Has sido atendido/a en los últimos dos años por algún/a enfermero/a?

- Sí
- No

¿Alguien de tu entorno cercano es enfermero/a?

- Sí
- No

En caso de haber respondido afirmativamente la respuesta anterior, matiza quién:

- Padre o madre o alguno de los/as abuelos/as.
- Hermano/a
- Tíos o resto de familiares
- Amigo/a cercano
- Otra...

¿Alguien de tu entorno cercano ha sido atendido/a en los últimos dos años por profesionales enfermeros/as?

- Sí
- No

En caso de haber respondido afirmativamente la respuesta anterior, matiza quién:

- [ ] Hermano/a
- [ ] Tíos o resto de familiares
- [ ] Amigo/a cercano
- [ ] Otra...

Después de la sección 2 Ir a la siguiente sección

Sección 3 de 3

CUESTIONARIO

Por favor, contesta con sinceridad a las siguientes cuestiones

01. Enfermería es un grado universitario en España

- Sí
- No
- No sé

02. Los/as enfermeros/as pueden acceder a estudios de postgrado (por ejemplo: máster, experto universitario...)

- Sí
- No
- No sé

Sí

No

No sé

04. Los/as enfermeros/as tienen acceso a especialidades a través de un proceso selectivo a nivel estatal (EIR)

Sí

No

No sé

05a. Consideras que los siguientes ámbitos son especialidades de enfermería: ENFERMERÍA EN SALUD MENTAL

Sí

No

No sé

05b. Consideras que los siguientes ámbitos son especialidades de enfermería: ENFERMERÍA DE PEDIATRÍA

Sí

No

No sé

05c. Consideras que los siguientes ámbitos son especialidades de enfermería: ENFERMERÍA

Sí

No

No sé

05d. Consideras que los siguientes ámbitos son especialidades de enfermería: ENFERMERÍA DE SALUD LABORAL

Sí

No

No sé

05e. Consideras que los siguientes ámbitos son especialidades de enfermería: ENFERMERÍA GERIÁTRICA

Sí

No

No sé

05f. Consideras que los siguientes ámbitos son especialidades de enfermería: MATRONA

Sí

No

No sé

05g. Consideras que los siguientes ámbitos son especialidades de enfermería: ENFERMERÍA DE CUIDADOS MEDICO-QUIRÚRGICOS

- No
- No sé

05h. Consideras que los siguientes ámbitos son especialidades de enfermería: ENFERMERÍA DE URGENCIAS Y EMERGENCIAS

- Sí
- No
- No sé

05i. Consideras que los siguientes ámbitos son especialidades de enfermería: ENFERMERÍA DE CUIDADOS INTENSIVOS

- Sí
- No
- No sé

A continuación, indica tu grado de acuerdo con las siguientes afirmaciones:

Valora del 0 al 5, donde:

0. Muy en desacuerdo
1. Un poco en desacuerdo
2. En desacuerdo
3. Un poco de acuerdo
4. De acuerdo

06. La enfermería es una profesión que puede ejercerse tanto por hombres como por mujeres

	0	1	2	3	4	5	
Muy en desacuerdo	○	○	○	○	○	○	Muy de acuerdo

07. La visibilidad social de los/as enfermeros/as es independiente del género de sus

	0	1	2	3	4	5	
Muy en desacuerdo	○	○	○	○	○	○	Muy de acuerdo

08. Proporcionalmente existen más hombres enfermeros que mujeres en puestos de

	0	1	2	3	4	5	
Muy en desacuerdo	○	○	○	○	○	○	Muy de acuerdo

09. Proporcionalmente existen más hombres que mujeres en puestos de docencia e investigación en cuidados

	0	1	2	3	4	5	
Muy en desacuerdo	○	○	○	○	○	○	Muy de acuerdo

10. Los/as enfermeros/as presentan autonomía en la toma de decisiones en los cuidados

	0	1	2	3	4	5	
Muy en desacuerdo	○	○	○	○	○	○	Muy de acuerdo

11. Los/as enfermeros/as son competentes para detectar o diagnosticar problemas de salud y necesidades de cuidados de la persona

	0	1	2	3	4	5	
Muy en desacuerdo	○	○	○	○	○	○	Muy de acuerdo

12. Los/as enfermeros/as pueden tratar de forma autónoma enfermedades leves de carácter autolimitado

	0	1	2	3	4	5	
Muy en desacuerdo	○	○	○	○	○	○	Muy de acuerdo

13. Los/as enfermeros/as están capacitadas para prescribir ("recetar") de forma autónoma medicamentos de venta libre

	0	1	2	3	4	5	
Muy en desacuerdo	○	○	○	○	○	○	Muy de acuerdo

14. Los/as enfermeros/as también trabajan de forma coordinada con otros miembros del equipo de salud para dar respuesta a las necesidades de los/as pacientes

	0	1	2	3	4	5	
Muy en desacuerdo	○	○	○	○	○	○	Muy de acuerdo

15. Los/as enfermeros/as asistenciales (es decir, que prestan atención directa a las personas) realizan tareas de gestión y administrativas en su práctica clínica diaria

	0	1	2	3	4	5	
Muy en desacuerdo	○	○	○	○	○	○	Muy de acuerdo

16. Cualquier profesional de la salud que esté capacitado/a debería poder tener acceso a un puesto de alta gestión dentro de una institución (por ejemplo, gerencia de una organización sanitaria, consejerías de salud...), independientemente de su profesión

0 1 2 3 4 5

17. Cualquier profesional de la salud que esté capacitado/a debería poder participar en el desarrollo de políticas sanitarias a cualquier nivel estatal, independientemente de ser hombre o

	0	1	2	3	4	5	
Muy en desacuerdo	○	○	○	○	○	○	Muy de acuerdo

18. La profesión enfermera es eminentemente científica

	0	1	2	3	4	5	
Muy en desacuerdo	○	○	○	○	○	○	Muy de acuerdo

19. Dentro de las competencias de los/as enfermeros/as se incluye la investigación

	0	1	2	3	4	5	
Muy en desacuerdo	○	○	○	○	○	○	Muy de acuerdo

20. Dentro de las publicaciones científicas las revistas enfocadas a cuidados presentan prestigio reconocido (factor de impacto)

	0	1	2	3	4	5	
Muy en desacuerdo	○	○	○	○	○	○	Muy de acuerdo

21. Los resultados de la investigación realizada por los/as enfermeros/as buscan el impacto en la mejora de la salud de las personas y de su comunidad

22. Los resultados de la investigación realizada por los/as enfermeros/as pueden promover el desarrollo de políticas sanitarias

	0	1	2	3	4	5	
Muy en desacuerdo	○	○	○	○	○	○	Muy de acuerdo

23. Los resultados de la investigación realizada por los/as enfermeros/as pueden contribuir a la sostenibilidad del sistema sanitario

	0	1	2	3	4	5	
Muy en desacuerdo	○	○	○	○	○	○	Muy de acuerdo

24. Dentro de las líneas de investigación realizadas por los/as enfermeros/as se encuentra la investigación en cuidados

	0	1	2	3	4	5	
Muy en desacuerdo	○	○	○	○	○	○	Muy de acuerdo

25. Dentro de las líneas de investigación realizadas por los/as enfermeros/as se encuentran los problemas de salud pública

	0	1	2	3	4	5	
Muy en desacuerdo	○	○	○	○	○	○	Muy de acuerdo

26. Dentro de las líneas de investigación realizadas por los/as enfermeros/as se encuentra las

	0	1	2	3	4	5	
Muy en desacuerdo	○	○	○	○	○	○	Muy de acuerdo

27. Son múltiples los posibles campos de investigación realizadas por los/as enfermeros/as

	0	1	2	3	4	5	
Muy en desacuerdo	○	○	○	○	○	○	Muy de acuerdo

28. Los/as enfermeros/as docentes desarrollan su competencia en el ámbito universitario

	0	1	2	3	4	5	
Muy en desacuerdo	○	○	○	○	○	○	Muy de acuerdo

29. Los/as enfermeros/as pueden ser profesores/as universitarios/as

	0	1	2	3	4	5	
Muy en desacuerdo	○	○	○	○	○	○	Muy de acuerdo

30. Los/as enfermeros/as desarrollan la competencia docente en el ámbito clínico

	0	1	2	3	4	5	
Muy en desacuerdo	○	○	○	○	○	○	Muy de acuerdo

31. Los/as enfermeros/as están presentes en los medios de comunicación como personas divulgadoras y educadoras en materia de salud

Muy en desacuerdo ○ ○ ○ ○ ○ ○ Muy de acuerdo

32. Los/as enfermeros/as tienen visibilidad en redes sociales como personas divulgadoras y educadoras en materia de salud

	0	1	2	3	4	5	
Muy en desacuerdo	○	○	○	○	○	○	Muy de acuerdo

33. Los medios de comunicación tienen relación estrecha con los colegios profesionales de enfermeros/as

	0	1	2	3	4	5	
Muy en desacuerdo	○	○	○	○	○	○	Muy de acuerdo

34. Los/as enfermeros/as son accesibles y están capacitados/as para dar respuesta inmediata a los medios de comunicación en situaciones de alarma y alerta sanitaria

	0	1	2	3	4	5	
Muy en desacuerdo	○	○	○	○	○	○	Muy de acuerdo

35. Los/as enfermeros/as proporcionan a los medios de comunicación información veraz y contrastada

	0	1	2	3	4	5	
Muy en desacuerdo	○	○	○	○	○	○	Muy de acuerdo

Muy en desacuerdo	○	○	○	○	○	○	Muy de acuerdo

37. Dentro de las competencias de los/as enfermeros/as se encuentra la promoción de la salud de la persona y de su comunidad

	0	1	2	3	4	5	
Muy en desacuerdo	○	○	○	○	○	○	Muy de acuerdo

38. Los/as enfermeros/as desarrollan un papel clave en la prevención de la enfermedad en la comunidad

	0	1	2	3	4	5	
Muy en desacuerdo	○	○	○	○	○	○	Muy de acuerdo

39. Los/as enfermeros/as desempeñan un papel fundamental en la comunidad a través del servicio telefónico de consejo sanitario

	0	1	2	3	4	5	
Muy en desacuerdo	○	○	○	○	○	○	Muy de acuerdo

40. La telemonitorización de los y las pacientes complejos es un ámbito de desarrollo de los/as enfermeros/as

	0	1	2	3	4	5	
Muy en desacuerdo	○	○	○	○	○	○	Muy de acuerdo

una correcta educación sanitaria

	0	1	2	3	4	5	
Muy en desacuerdo	○	○	○	○	○	○	Muy de acuerdo

42. Los/as enfermeros/as son buenos agentes de divulgación sanitaria

	0	1	2	3	4	5	
Muy en desacuerdo	○	○	○	○	○	○	Muy de acuerdo

43. Conoces a través de los medios de comunicación convencionales aspectos de la actividad de la profesión de enfermería

	0	1	2	3	4	5	
Muy en desacuerdo	○	○	○	○	○	○	Muy de acuerdo

44. Los resultados de la investigación en enfermería se comparten también con la sociedad a través de los medios de comunicación

	0	1	2	3	4	5	
Muy en desacuerdo	○	○	○	○	○	○	Muy de acuerdo

Respecto a la actual pandemia de Covid-19...

Descripción (opcional)

45. Tu percepción de los/as enfermeros/as ha mejorado tras la pandemia Covid-

46. La labor gestora los/as enfermeros/as en la resolución de la pandemia Covid-19 ha sido determinante

	0	1	2	3	4	5	
Muy en desacuerdo	○	○	○	○	○	○	Muy de acuerdo

47. La crisis de la pandemia Covid-19 ha contribuido a visibilizar las competencias investigadoras de los/as enfermeros/as

	0	1	2	3	4	5	
Muy en desacuerdo	○	○	○	○	○	○	Muy de acuerdo

48. En la comisión de reconstrucción de la pandemia Covid-19 debería existir una figura

	0	1	2	3	4	5	
Muy en desacuerdo	○	○	○	○	○	○	Muy de acuerdo

Muchas gracias / Eskerik asko

Descripción (opcional)
