# Supplementary material for: A questionnaire development to assess the social representation of nurses in the Basque Country: a psychometric assessment
Source: PeerJ. 2022 Sep 23;10:e13903. doi: 10.7717/peerj.13903 (PMC9512000; doi:10.7717/peerj.13903)
Supplement: Supplemental Information 3 [file peerj-10-13903-s003.docx]

SOCIAL REPRESENTATION OF NURSES BY JOURNALISM STUDENTS

Thank you very much for taking part in this survey. Next, we will ask you several questions that aim to explore the social representation of nursing from the perspective of students studying for the Degree in Journalism.

This survey is part of the research project NURSES FROM THE PERSPECTIVE OF UNDERGRADUATE STUDENTS OF JOURNALISM IN THE BASQUE COUNTRY, which has had a favourable report from the Ethics Committee of the University of the Basque Country (UPV/EHU). The main researchers of this study are: Verónica Tíscar (veronica.tiscargonzales@osakidetza.eus), Leire Iturregui (leire.iturregui@ehu.eus), Eztizen Miranda ([eztizen.miranda@ehu.eus](mailto:eztizen.miranda@ehu.eus)), Sendoa Ballesteros ([sendoa.ballesteros@ehu.eus](mailto:sendoa.ballesteros@ehu.eus)) and Maria Ángeles Cidoncha ([mariaangeles.cidonchamoreno@osakidetza.eus](mailto:mariaangeles.cidonchamoreno@osakidetza.eus))

The survey is completely ANONYMOUS and VOLUNTARY, so please be as honest as possible with your answers. It will only take you 10 minutes to complete.

By completing it voluntarily, we understand that you give your tacit consent to participate in the research. Remember, that once the survey has started, you can withdraw and leave at any time, if you wish to do so.

your socio-demographic data

1. Gender

Mark only one circle

- Male
- Female
- Non-binary

1. Date of birth
2. Previous studies

Mark only one circle

- - High School
  - Professional training
  - Other degree or equivalent studies
  - Others

1. Current academic year

Mark only one circle

- - 1^st^
  - 2^nd^
  - 3^th^
  - 4^th^

1. Have you been cared for by a nurse in the last two years?

Mark only one circle

- - Yes
  - No

1. Is someone close to you a nurse?

Mark only one circle

- Yes
- No

1. If you answered yes to the previous question, please specify who:

Select all that apply

- Parent or grandparents
- Siblings
- Aunts, uncles or other relatives
- Close friend
- Other ­­­­­­­­­­­­­­­­­­­­­­­________________________

1. Have nursing professionals attended anyone in your immediate circle in the last two years?

Mark only one circle

- - Yes
  - No

1. If you answered yes to the previous question, please specify who:

Select all that apply

- Parent or grandparents
- Siblings
- Aunts, uncles or other relatives
- Close friend
- Other ­­­­­­­­­­­­­­­­­­­­­­­________________________

QUESTIONNAIRE

Please answer the following questions honestly.

01. Nursing is a university degree in Spain

Mark only one circle

- Yes
- No
- I don´t know

02. Nurses can access postgraduate studies (e.g Master's degree, PhD...)

Mark only one circle

- Yes
- No
- I don´t know

03. Nurses are eligible to undertake doctoral studies

Mark only one circle

- Yes
- No
- I don´t know

04. Nurses have access to specialities through a selective process, at state level, called the Resident Nurse Intern (EIR in Spanish)

Mark only one circle

- Yes
- No
- I don´t know

05a. You consider the following area to be a nursing speciality:

MENTAL HEALTH NURSING

Mark only one circle

- Yes
- No
- I don´t know

05b. You consider the following area to be a nursing speciality:

PAEDIATRIC NURSING

Mark only one circle

- Yes
- No
- I don´t know

05c. You consider the following area to be a nursing speciality:

FAMILY AND COMMUNITY NURSING

Mark only one circle

- Yes
- No
- I don´t know

05d. You consider the following area to be a nursing speciality:

OCCUPATIONAL HEALTH NURSING

Mark only one circle

- Yes
- No
- I don´t know

05e. You consider the following area to be a nursing speciality:

GERIATRIC NURSING

Mark only one circle

- Yes
- No
- I don´t know

05f. You consider the following area to be a nursing speciality:

MIDWIFE

Mark only one circle

- Yes
- No
- I don´t know

05g. You consider the following area to be a nursing speciality:

MEDICAL-SURGICAL NURSING CARE

Mark only one circle

- Yes
- No
- I don´t know

05h. You consider the following area to be a nursing speciality:

EMERGENCY AND URGENCY NURSING

Mark only one circle

- Yes
- No
- I don´t know

05i. You consider the following area to be a nursing speciality:

INTENSIVE CARE NURSING

Mark only one circle

- Yes
- No
- I don´t know

Please indicate your level of agreement with the following statements:

Rate from 0 to 5:

0. Strongly disagree

1. Slightly disagree

2. Disagree

3. Somewhat agree

4. Agree

5. Strongly agree

06. Nursing is a profession that can be practised by both men and women.

Mark only one square

|  | 0 | 1 | 2 | 3 | 4 | 5 |  |
| --- | --- | --- | --- | --- | --- | --- | --- |
| Strongly disagree |  |  |  |  |  |  | Strongly agree |

07. The social visibility of nurses is independent of the gender of the profession.

Mark only one square

|  | 0 | 1 | 2 | 3 | 4 | 5 |  |
| --- | --- | --- | --- | --- | --- | --- | --- |
| Strongly disagree |  |  |  |  |  |  | Strongly agree |

08. Proportionally, there are more male nurses than female nurses in senior positions of responsibility.

Mark only one square

|  | 0 | 1 | 2 | 3 | 4 | 5 |  |
| --- | --- | --- | --- | --- | --- | --- | --- |
| Strongly disagree |  |  |  |  |  |  | Strongly agree |

09. Proportionally, there are more men than women in teaching and research positions in care.

Mark only one square

|  | 0 | 1 | 2 | 3 | 4 | 5 |  |
| --- | --- | --- | --- | --- | --- | --- | --- |
| Strongly disagree |  |  |  |  |  |  | Strongly agree |

10. Nurses have autonomy in decision-making about the care of their patients.

Mark only one square

|  | 0 | 1 | 2 | 3 | 4 | 5 |  |
| --- | --- | --- | --- | --- | --- | --- | --- |
| Strongly disagree |  |  |  |  |  |  | Strongly agree |

11. Nurses are competent to detect or diagnose the health problems and care needs of individuals.

Mark only one square

|  | 0 | 1 | 2 | 3 | 4 | 5 |  |
| --- | --- | --- | --- | --- | --- | --- | --- |
| Strongly disagree |  |  |  |  |  |  | Strongly agree |

12. Nurses can autonomously treat minor illnesses of a self-limiting nature.

Mark only one square

|  | 0 | 1 | 2 | 3 | 4 | 5 |  |
| --- | --- | --- | --- | --- | --- | --- | --- |
| Strongly disagree |  |  |  |  |  |  | Strongly agree |

13. Nurses are able to independently prescribe over-the-counter medicines.

Mark only one square

|  | 0 | 1 | 2 | 3 | 4 | 5 |  |
| --- | --- | --- | --- | --- | --- | --- | --- |
| Strongly disagree |  |  |  |  |  |  | Strongly agree |

14. Nurses also work in coordination with other members of the health team to respond to the needs of patients.

Mark only one square

|  | 0 | 1 | 2 | 3 | 4 | 5 |  |
| --- | --- | --- | --- | --- | --- | --- | --- |
| Strongly disagree |  |  |  |  |  |  | Strongly agree |

15. Nursing assistants (i.e. nurses who provide direct care to people) perform managerial and administrative tasks in their daily clinical practice.

Mark only one square

|  | 0 | 1 | 2 | 3 | 4 | 5 |  |
| --- | --- | --- | --- | --- | --- | --- | --- |
| Strongly disagree |  |  |  |  |  |  | Strongly agree |

16. Any trained health professional should be able to have access to a senior management position within an institution (e.g., management of a health organisation, health councils...) regardless of their profession.

Mark only one square

|  | 0 | 1 | 2 | 3 | 4 | 5 |  |
| --- | --- | --- | --- | --- | --- | --- | --- |
| Strongly disagree |  |  |  |  |  |  | Strongly agree |

17. Any trained health professional should be able to participate in the development of health policies at any state level, regardless of whether they are male or female.

Mark only one square

|  | 0 | 1 | 2 | 3 | 4 | 5 |  |
| --- | --- | --- | --- | --- | --- | --- | --- |
| Strongly disagree |  |  |  |  |  |  | Strongly agree |

18. The nursing profession is eminently scientific.

Mark only one square

|  | 0 | 1 | 2 | 3 | 4 | 5 |  |
| --- | --- | --- | --- | --- | --- | --- | --- |
| Strongly disagree |  |  |  |  |  |  | Strongly agree |

19. Research is part of the competencies of nurses.

Mark only one square

|  | 0 | 1 | 2 | 3 | 4 | 5 |  |
| --- | --- | --- | --- | --- | --- | --- | --- |
| Strongly disagree |  |  |  |  |  |  | Strongly agree |

20, Journals focused on care are recognised for their prestige among scientific publications (impact factor).

Mark only one square

|  | 0 | 1 | 2 | 3 | 4 | 5 |  |
| --- | --- | --- | --- | --- | --- | --- | --- |
| Strongly disagree |  |  |  |  |  |  | Strongly agree |

21. The results of research carried out by nurses aim to improve the health of individuals and their communities.

Mark only one square

|  | 0 | 1 | 2 | 3 | 4 | 5 |  |
| --- | --- | --- | --- | --- | --- | --- | --- |
| Strongly disagree |  |  |  |  |  |  | Strongly agree |

22. The results of research conducted by nurses can promote the development of health policies.

Mark only one square

|  | 0 | 1 | 2 | 3 | 4 | 5 |  |
| --- | --- | --- | --- | --- | --- | --- | --- |
| Strongly disagree |  |  |  |  |  |  | Strongly agree |

23. The results of research conducted by nurses can contribute to the sustainability of the healthcare system.

Mark only one square

|  | 0 | 1 | 2 | 3 | 4 | 5 |  |
| --- | --- | --- | --- | --- | --- | --- | --- |
| Strongly disagree |  |  |  |  |  |  | Strongly agree |

24. The research carried out by nurses include the field of healthcare.

Mark only one square

|  | 0 | 1 | 2 | 3 | 4 | 5 |  |
| --- | --- | --- | --- | --- | --- | --- | --- |
| Strongly disagree |  |  |  |  |  |  | Strongly agree |

25. The research carried out by nurses include public health problems.

Mark only one square

|  | 0 | 1 | 2 | 3 | 4 | 5 |  |
| --- | --- | --- | --- | --- | --- | --- | --- |
| Strongly disagree |  |  |  |  |  |  | Strongly agree |

26. The research carried out by nurses include gender inequalities and social determinants of health.

Mark only one square

|  | 0 | 1 | 2 | 3 | 4 | 5 |  |
| --- | --- | --- | --- | --- | --- | --- | --- |
| Strongly disagree |  |  |  |  |  |  | Strongly agree |

27. Nurses can carry out research into many possible fields of research.

Mark only one square

|  | 0 | 1 | 2 | 3 | 4 | 5 |  |
| --- | --- | --- | --- | --- | --- | --- | --- |
| Strongly disagree |  |  |  |  |  |  | Strongly agree |

28. Nurse educators develop their competence at university level.

Mark only one square

|  | 0 | 1 | 2 | 3 | 4 | 5 |  |
| --- | --- | --- | --- | --- | --- | --- | --- |
| Strongly disagree |  |  |  |  |  |  | Strongly agree |

29. Nurses can become university lecturers.

Mark only one square

|  | 0 | 1 | 2 | 3 | 4 | 5 |  |
| --- | --- | --- | --- | --- | --- | --- | --- |
| Strongly disagree |  |  |  |  |  |  | Strongly agree |

30. Nurses develop teaching competencies in clinical settings.

Mark only one square

|  | 0 | 1 | 2 | 3 | 4 | 5 |  |
| --- | --- | --- | --- | --- | --- | --- | --- |
| Strongly disagree |  |  |  |  |  |  | Strongly agree |

31. Nurses engage with the media as health educators and communicators.

Mark only one square

|  | 0 | 1 | 2 | 3 | 4 | 5 |  |
| --- | --- | --- | --- | --- | --- | --- | --- |
| Strongly disagree |  |  |  |  |  |  | Strongly agree |

32. Nurses are visible in social networks as disseminators and educators in health matters.

Mark only one square

|  | 0 | 1 | 2 | 3 | 4 | 5 |  |
| --- | --- | --- | --- | --- | --- | --- | --- |
| Strongly disagree |  |  |  |  |  |  | Strongly agree |

33. The media has a close relationship with the professional nursing associations.

Mark only one square

|  | 0 | 1 | 2 | 3 | 4 | 5 |  |
| --- | --- | --- | --- | --- | --- | --- | --- |
| Strongly disagree |  |  |  |  |  |  | Strongly agree |

34. Nurses are accessible and trained to respond immediately to the media when there are emergencies and health alerts.

Mark only one square

|  | 0 | 1 | 2 | 3 | 4 | 5 |  |
| --- | --- | --- | --- | --- | --- | --- | --- |
| Strongly disagree |  |  |  |  |  |  | Strongly agree |

35. Nurses provide the media with accurate and verified sources of information.

Mark only one square

|  | 0 | 1 | 2 | 3 | 4 | 5 |  |
| --- | --- | --- | --- | --- | --- | --- | --- |
| Strongly disagree |  |  |  |  |  |  | Strongly agree |

36. The competences of nurses are known at the societal level.

Mark only one square

|  | 0 | 1 | 2 | 3 | 4 | 5 |  |
| --- | --- | --- | --- | --- | --- | --- | --- |
| Strongly disagree |  |  |  |  |  |  | Strongly agree |

37. The competencies of nurses include promoting the health of individuals and his or her community.

Mark only one square

|  | 0 | 1 | 2 | 3 | 4 | 5 |  |
| --- | --- | --- | --- | --- | --- | --- | --- |
| Strongly disagree |  |  |  |  |  |  | Strongly agree |

38. Nurses play a key role in preventing disease in the community.

Mark only one square

|  | 0 | 1 | 2 | 3 | 4 | 5 |  |
| --- | --- | --- | --- | --- | --- | --- | --- |
| Strongly disagree |  |  |  |  |  |  | Strongly agree |

39. Nurses play a key role in the community by providing telephone health advice services.

Mark only one square

|  | 0 | 1 | 2 | 3 | 4 | 5 |  |
| --- | --- | --- | --- | --- | --- | --- | --- |
| Strongly disagree |  |  |  |  |  |  | Strongly agree |

40. Telemonitoring of complex patients is an area of development for nurses.

Mark only one square

|  | 0 | 1 | 2 | 3 | 4 | 5 |  |
| --- | --- | --- | --- | --- | --- | --- | --- |
| Strongly disagree |  |  |  |  |  |  | Strongly agree |

41. Nurses are trained and knowledgeable professionals who can ensure people have proper health education.

Mark only one square

|  | 0 | 1 | 2 | 3 | 4 | 5 |  |
| --- | --- | --- | --- | --- | --- | --- | --- |
| Strongly disagree |  |  |  |  |  |  | Strongly agree |

42. Nurses are good health outreach workers.

Mark only one square

|  | 0 | 1 | 2 | 3 | 4 | 5 |  |
| --- | --- | --- | --- | --- | --- | --- | --- |
| Strongly disagree |  |  |  |  |  |  | Strongly agree |

43. You are familiar with aspects of the nursing profession through traditional media.

Mark only one square

|  | 0 | 1 | 2 | 3 | 4 | 5 |  |
| --- | --- | --- | --- | --- | --- | --- | --- |
| Strongly disagree |  |  |  |  |  |  | Strongly agree |

44. The results of nursing research are also shared with society through the media.

Mark only one square

|  | 0 | 1 | 2 | 3 | 4 | 5 |  |
| --- | --- | --- | --- | --- | --- | --- | --- |
| Strongly disagree |  |  |  |  |  |  | Strongly agree |

Regarding the current COVIDd-19 pandemic:

45. Your perception of nurses has improved after the COVID-19 pandemic.

Mark only one square

|  | 0 | 1 | 2 | 3 | 4 | 5 |  |
| --- | --- | --- | --- | --- | --- | --- | --- |
| Strongly disagree |  |  |  |  |  |  | Strongly agree |

46. Nurses have shown decisive management in helping to resolve the COVID-19 pandemic.

Mark only one square

|  | 0 | 1 | 2 | 3 | 4 | 5 |  |
| --- | --- | --- | --- | --- | --- | --- | --- |
| Strongly disagree |  |  |  |  |  |  | Strongly agree |

47. The COVID-19 pandemic crisis has contributed to raising the visibility of the research competencies of nurses.

Mark only one square

|  | 0 | 1 | 2 | 3 | 4 | 5 |  |
| --- | --- | --- | --- | --- | --- | --- | --- |
| Strongly disagree |  |  |  |  |  |  | Strongly agree |

48. There should be a nurse figure in the COVID-19 pandemic reconstruction commission.

Mark only one square

|  | 0 | 1 | 2 | 3 | 4 | 5 |  |
| --- | --- | --- | --- | --- | --- | --- | --- |
| Strongly disagree |  |  |  |  |  |  | Strongly agree |

Thank you very much
